# Cancer-specific survival by stage of bladder cancer and factors collected by Mallorca Cancer Registry associated to survival

**DOI:** 10.1186/s12885-021-08418-y

**Published:** 2021-06-07

**Authors:** J. Ripoll, M. Ramos, J. Montaño, J. Pons, A. Ameijide, P. Franch

**Affiliations:** 1 Primary Care Research Unit of Mallorca, Balearic Health Service, Palma, Spain; 2 Balearic Islands Health Research Institute (IdISBa), Palma de Mallorca, 07120 Illes Balears, Spain; 3 Mallorca Cancer Registry, Balearic Islands Public Health Department, Palma, Spain; 4 University of the Balearic Islands, Palma, Spain; 5 Tarragona Cancer Registry, Cancer Epidemiology and Prevention Service. Sant Joan de Reus University Hospital, IISPV., Reus, Spain

**Keywords:** Bladder neoplasms, Survival, Stage, Multiple imputation

## Abstract

**Background:**

Information about survival by stage in bladder cancer is scarce, as well as about survival of non-invasive bladder cancer. The aims of this study are: 1) to find out the distribution of bladder cancer by stage; 2) to determine cancer-specific survival by stage of bladder cancer; 3) to identify factors that explain and predict the likelihood of survival and the risk of dying from these cancers.

**Methods:**

Incident bladder cancer cases diagnosed between 2006 and 2011 were identified through the Mallorca Cancer Registry. Inclusion criteria: cases with code C67 according to the ICD-O 3rd edition with any behaviour and any histology, except lymphomas and small cell carcinomas. Cases identified exclusively through the death certificate were excluded. We collected the following data: sex; age; date and method of diagnosis; histology according to the ICD-O 3rd edition; T, N, M and stage at the time of diagnosis; and date of follow-up or death. End point of follow-up was 31 December 2015. Multiple imputation (MI) was performed to estimate cases with unknown stage. Cases with benign or indeterminate behaviour were excluded for the survival analysis. Actuarial and Kaplan-Meier methods and Cox regression models were used for survival analysis.

**Results:**

One thousand nine hundred fourteen cases were identified. 14% were women and 65.4% were 65 years or older. 3.9% had no stage (benign or undetermined behaviour) and 11.5% had unknown stage. After MI, 37.5% were in stage Ta (non-invasive papillary carcinoma), 3.2% in stage Tis (carcinoma in situ), 34.3% in stage I, 11.7% in Stage II, 4.3% in stage III, and 9.0% in stage IV. Survival was 76% at 5 years. Survival by stage: 98% at stage Ta, 90% at stage Tis, 85% at stage I, 45% at stage II, 35% at stage III, and 7% at stage IV. The Cox model showed that age, histology, and stage, but not sex, were associated with survival.

**Conclusion:**

Bladder cancer survival vary greatly with stage, among both non-invasive and invasive cases. The percentage of non-invasive cancers is high. Stage, age, and histology are associated to survival.

## Background

Bladder cancer is the second most frequent genitourinary cancer after prostate cancer. Europe has one of the highest bladder cancer incidences, especially in Italy and Spain [1]. According to the Spanish Network of Cancer Registries (REDECAN), bladder cancer is the third most frequent cancer in men and the seventh in women, considering colon and rectal cancer separately. Estimated world adjusted incidence rates for 2019 were 37.7 (CI at 95%: 33.6–42.3) by 100,000 habitants in men and 7.4 (5.6–9.6) in women [2]. In 2015, adjusted mortality rates were 10.46 by 100,000 habitants in men and 1.71 in women [3].

The EUROCARE-5 study estimated, for the period 2000–2007, a relative survival at 5 years for bladder cancer of 70.4% (69.3–71.4) for Spain; slightly higher than the European average, which was 68.6% (68.3–68.9). A huge variability in bladder cancer survival was observed due to the inclusion or not of non-invasive cases [4].

Stage at diagnosis is the most important prognostic factor for invasive bladder cancer, while grade is the most important prognostic factor for non-invasive bladder cancer [5]. Regarding stage, most studies use the simplified classification based on: localized, regional, and distant categories [6–8]. Information about survival by stage in bladder cancer according to the TNM system is scarce [9], as well as about survival of non-invasive bladder cancer [8]. Clinicians use the classification based on: non-muscle invasive bladder cancer (NMIBC), including in situ carcinomas and T1, and muscle-invasive bladder cancer (MIBC), including T2–T4 tumours. Even with optimal treatment, bladder cancer recurs in more than 50% of cases of NMIBC and can progress to MIBC in up to 20% of patients [10].

One of the problems that face population-based cancer registries collecting stage is missing values; the handling of which becomes a challenge in epidemiological research because it introduces bias. Multiple imputation (MI) solves bias and underestimation of population variability by offering similar estimates to the ones obtained with complete data [11], and it is an appropriate method to handle missing values of stage in survival cancer studies [12].

Having information about distribution of cases by stage and survival by stage in bladder cancer is useful for the monitoring of survival trends, and as an overall measure of the effectiveness of health care system in cancer prevention, early diagnosis, and treatment.

The aims of this study were: 1) to find out the distribution of bladder and urinary tract cancer by stage; 2) to determine cancer-specific survival by stage of bladder cancer; 3) to identify factors that explain and predict the likelihood of survival and the risk of dying from this cancer.

## Methods

Retrospective follow-up study of patients living in Mallorca diagnosed with bladder cancer between 2006 and 2011, identified through the Mallorca Cancer Registry.

Study population: cases with code C67 according to the ICD-O 3rd edition with any behaviour and histology except lymphomas (from 9590 to 9720 both included) and small cell carcinomas (from 8041 to 8045 both included) were included, while cases identified exclusively through the death certificate (DCO cases) were excluded.

IACR/IARC rules for multiple cancers were used [13]. Thus, only the first cancer was registered, whether it was uncertain behaviour, in situ, or invasive. If, subsequently, there was a progression from non-invasive to invasive, the first registered cancer was not modified.

The following data were collected: sex, age, diagnostic method; histology and behaviour according to the ICD-O 3rd edition [13]; date of diagnosis; pathological or clinical tumour size (T), pathological or clinical regional lymph nodes (N), metastasis (M) and stage; date of last follow-up or date of death, and cause of death (bladder cancer or other causes).

Age was grouped as: 15–44 years old, 45–54, 55–64, 65–74, and 75 and over. Diagnostic method was recorded as clinical, pathological, or unknown. Histology was recorded as: papillary transitional cell neoplasia (8130), solid transitional cell neoplasia (8120), and other histology and unspecified (8000, 8001, 8010, 8020, 8033, 8070, 8071, 8082, 8140, 8310, 8480, 8490, 8255, 8900). Behaviour was registered as uncertain, in situ, and invasive.

Stage was calculated according to the UICC 7th edition [14], but regrouped in the following categories: Ta, Tis, I, II, III, IV, no stage (uncertain behaviour). Pathological T or N status was prioritised over clinical. An integrated approach [14] was used by combining pathological and clinical components to obtain the stage. The clinical records of cases with missing stage were reviewed in depth to minimise the number of lost values. We did the following assumptions: if T was 1 and N and M were missing, we assigned stage 1; if T was 2 and N and M were missing, we assigned stage 2, as some authors recommend for prostate cancer [15].

Time was calculated from date of diagnosis to date of death or date of the last follow-up. Vital status referred to the state (alive or dead from bladder cancer or from other causes) at the time of the last follow-up. The clinical records of deceased cases were reviewed in depth to establish precisely the cause of death. Cases that emigrated from Mallorca and lost cases were censored, as well as deaths from other causes for cancer-specific survival. The starting point of follow-up was the date of diagnosis, and the end point was 31 December 2015.

### Statistical analysis

MI was used to obtain stage when this was unknown, following three main steps [16]. First, we ran the imputation model and replaced each missing value with a set of five imputations by applying the multiple imputation chained equation (MICE) procedure. We made the imputation using the variables sex, age, histology, vital status and survival time. Secondly, we analysed the resulting five imputed and complete data sets independently by applying the Cox regression model. Finally, we obtained a single Cox model using Rubin’s rules [17] to combine the five estimates resulting from the previous Cox regression model. A more detailed description about the MICE procedure can be found in Ramos et al. [18].

We applied the cause-specific survival analysis developed by actuarial and Kaplan-Meier methods to estimate likelihood of survival and risk of death; relative survival using the Ederer II method [19]; the log-rank test to evaluate the statistical differences of the observed survival curves by each categorical variable; the log-rank test for trend to analyze the type of trend of the two variables that can be considered as ordinal, age groups and stage; we also calculated people at risk at the beginning of the study, at 3 and at 5 years. Finally, the Cox regression models were developed to identify prognostic factors of the risk of death. Cases with uncertain behaviour were excluded for the survival analysis, since they have no stage, our main study variable. We considered age as a continuous variable because our interest was to know the effect of each unit increase on the risk of dying from bladder or urinary tract cancer. The proportional hazard assumption for each covariate was tested by introducing time dependent variables. Since age and histology did not meet this assumption, we applied the extended Cox regression, which not only analyses the effect of covariates on the risk of dying, but also allows for the modelling of the time dependent effect of age and histology covariates. The procedure for selecting the variables in the final Cox model was based on the likelihood ratio (LR)test. Thus, initially, sex, age, histology and stage were introduced into the model, as well as time-dependent variables of age and histology. To compare the effect of the imputation procedure on the hazard ratio estimation of covariates, the extended Cox regression was performed before and after MI.

MI was carried out with STATA 13, cancer-specific survival analysis with SPSS 23 and relative survival with the “relsurv” library of R.

## Results

A total of 2060 cases of bladder cancer were identified between 2006 and 2011. We worked with 1914 cases because 10 DCO, 1 lymphoma, 12 small cell carcinomas, and 22 cases without follow up data were excluded. Of the 1914 cases, only 14% were women and 65.4% were 65 years or older. 96.3% were diagnosed by pathological methods and There were 11.5% of cases with unknown stage. After MI, 37.5% were in stage Ta (non invasive papillary carcinoma), 3.2% in stage Tis (carcinoma in situ), 34.3% in stage I, 11.7% in stage II, 4.3% in stage III, and 9.01% in stage IV. Almost three of four cases (76.7%) were NMIBC. Full description of the sample is presented in Table 1.

**Table 1:** Sociodemographic and clinical description of bladder cancer cases diagnosed in Mallorca between 2006 and 2011 (*N* = 1914)

Variable	Categories	N	%	% Valid	After MI
**Sex**	Women	268	14.0	14.0	
	Men	1646	86.0	86.0	
**Age**	15–44	45	2.4	2.4	
	45–54	172	9.0	9.0	
	55–64	445	23.2	23.2	
	65–74	548	30.2	30.2	
	75 or +	674	35.2	35.2	
**Diagnostic method**	Pathological	1843	96.3	96.3	
	Clinical	65	3.4	3.4	
	Unknown	6	0.3	0.3	
**Histology**	Papillary transitional neoplasia	1096	57.3	57.3	
	Solid transitional neoplasia	713	37.3	37.3	
	Other histology and unspecified	105	5.5	5.5	
**Behaviour**	Invasive	1160	60.6	60.6	
	In situ	680	35.5	35.5	
	Uncertain	74	3.9	3.9	
**Clinical or pathological tumour size (T_PT)**	1	566	29.6	44.0	
	2	207	10.8	16.1	
	3	68	3.6	5.3	
	4a	58	3.0	4.5	
	4b	1	0.1	0.1	
	a (histology 8130 and behaviour in situ)	298	15.6	23.2	
	is (histology 8120 and behaviour in situ)	13	0.7	1.0	
	uncertain behaviour	74	3.9	5.8	
	Missing	629	32.9		
**Clinical or pathological regional lymph nodes (N_PN)**	0	187	8.8	68.8	
	1	33	1.7	12.1	
	2	46	2.4	16.9	
	3	6	0.3	2.2	
	Missing	1642	85.8		
**Metastasis(M)**	0	237	12.4	80.6	
	1	57	3.0	19.4	
	Missing	1620	84.6		
**Stage**^**a**^	Ta	625	32.7	38.6	37.3
	Tis	53	2.8	3.3	3.2
	I	564	29.5	34.8	34.2
	II	181	9.5	11.2	11.8
	III	67	3.5	4.1	4.3
	IV	129	6.7	8.0	9.1
	No stage	74	3.9		
	Missing	221	11.5		

^a^values imputed for each category of stage: Ta: 306 (27.7%); Tis: 27 (2.4%); I: 326 (29.5%); II: 184 (16.6%); III: 66 (5.9%); IV: 196 (17.7%)

Survival analysis was performed with 1840 cases, since cases with uncertain behaviour were excluded. Mean time of survival was 6.4 years. Cancer-specific survival was 88% 1 year after diagnosis, 80% at 3 years, 76% at 5 years, and seemed to stabilise 7 years after diagnosis (Table 2). Relative survival was 87% 1 year after diagnosis, 77% at 3 years and 69% at 5 years. After MI, cancer-specific survival rates at 5 years after diagnosis were: 98% for stage Ta, 90% for stage Tis, 85% for stage I, 45% for stage II, 35% for stage III, and 7% for stage IV. Without MI, survival would have been a little overestimated in non-invasive and invasive stages, as it is shown in Table 2 (99% for stage Ta, 91% for stage Tis, 86% for stage I, 48% for stage II, 37% for stage III or 8% for stage IV). After MI, relative survival rates 5 years after diagnosis were: 91% for stage Ta, 82% for stage Tis, 76% for stage I, 42% for stage II, 26% for stage III and 7% for stage IV.

**Table 2:** Cancer-specific survival rates of bladder cancer cases diagnosed in Mallorca between 2006 and 2011 by actuarial method by follow-up year in percentages

Original data set ***n*** = 1619								Imputed data set ***n*** = 1840						
Year	Stage Ta	Stage Tis	Stage I	Stage II	Stage III	Stage IV	Total	Stage Ta	Stage Tis	Stage I	Stage II	Stage III	Stage IV	Total
1	100	98	97	74	66	44	88	100	98	96	71	65	42	88
2	99	98	95	59	49	27	83	99	97	94	57	46	25	83
3	99	94	91	55	45	19	80	99	93	90	52	42	18	80
4	99	91	89	49	45	12	78	99	90	88	47	41	11	78
5	99	91	86	48	37	8	76	98	90	85	45	35	7	76
6	98	85	85	46	37	8	75	98	85	84	43	35	6	75
7	97	85	85	46	37	8	74	97	85	84	43	35	6	74
8	97	85	85	46	37	8	74	97	85	84	43	35	6	74

**Fig. 1:**
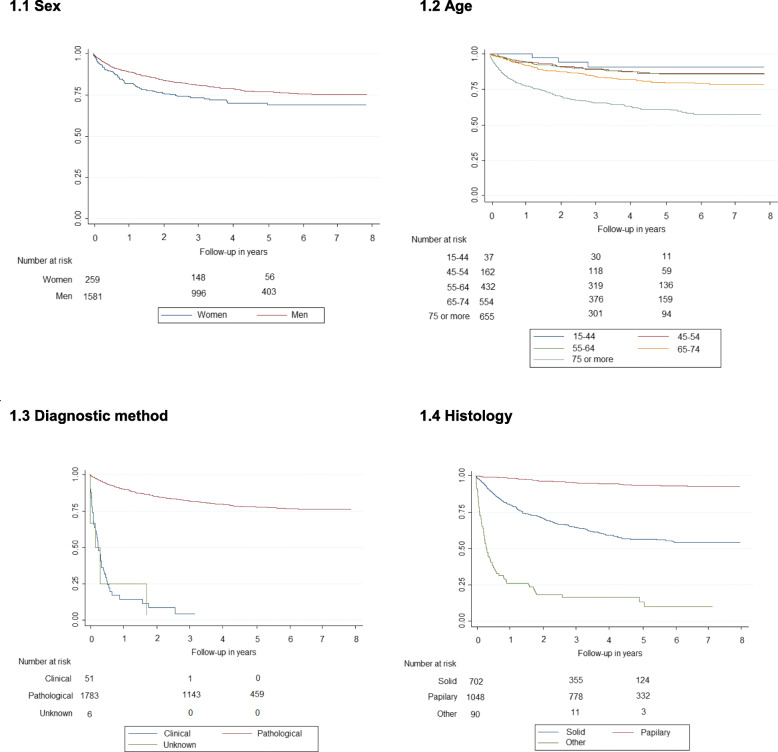
Survival of bladder cancer cases diagnosed in Mallorca between 2006 and 2011 by sex, age, diagnostic method and histology

**Fig. 2:**
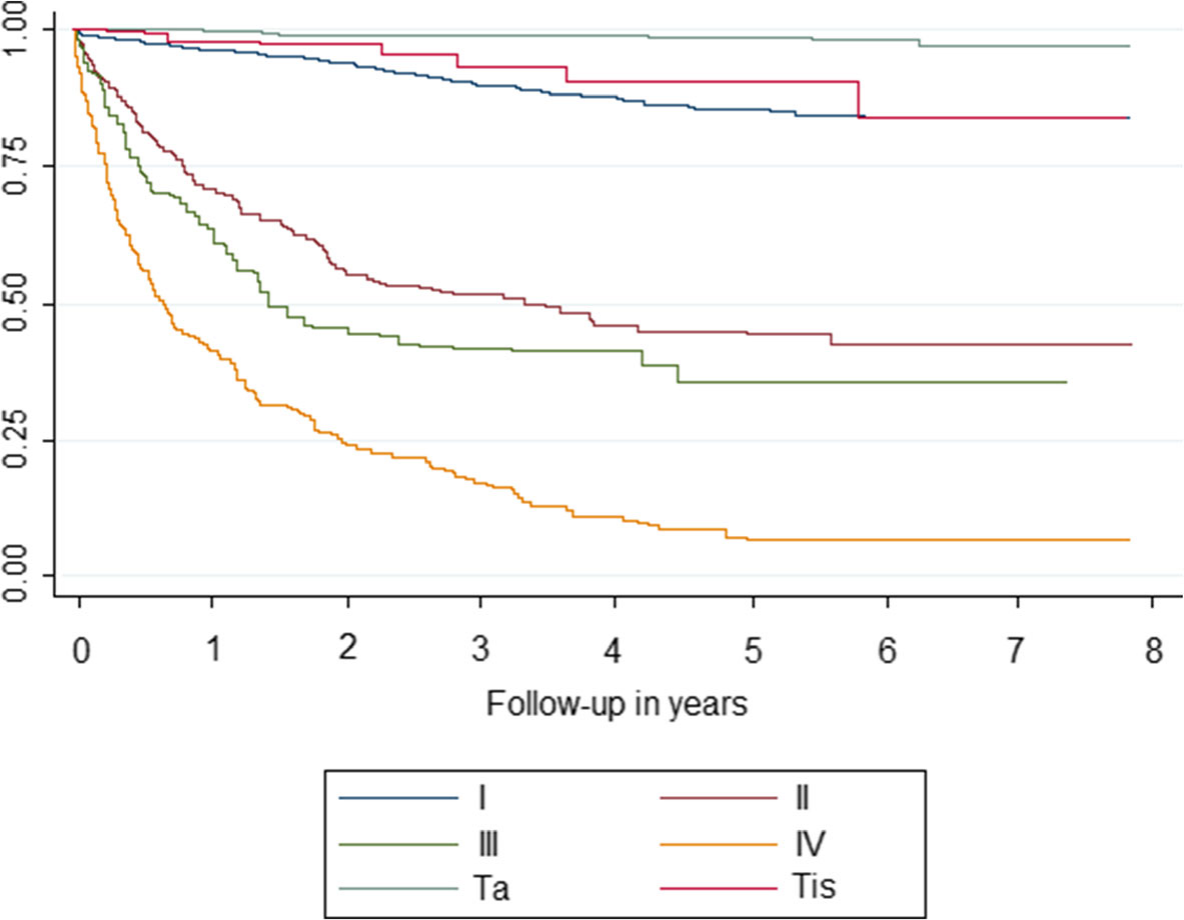
Survival by stage of bladder cancer cases diagnosed in Mallorca between 2006 and 2011 after multiple imputation

Survival curves showed differences in bladder cancer survival by sex (*p* < 0.001), age (*p* < 0.001), method of diagnosis (*p* < 0.001), histology (*p* < 0.001) (Fig. 1), and stage (*p* < 0.001) (Fig. 2). Comparing each variable by pair of categories, the group of 75 and older had worse survival, while papillary transitional cell carcinoma presented better survival. Stage Ta had better survival; and no differences were observed between stage Tis and stage I, and between stage II and stage III. Trend analysis shows that age and stage have a significant linear trend (*p* < 0.001).

The maximum likelihood criterion included age, histology, and stage in the final Cox model, but we decided to include also sex according to the bibliographic review. Therefore, time-dependent variables of age and histology were excluded. Table 3 shows the Cox model before and after MI. Both models (original vs. MI model) determined that younger cases, patients with papillary transitional cell carcinoma, and patients diagnosed in stage Tis and stage I, have a better prognosis. From stage I, survival of bladder cancer worsened greatly.

**Table 3:** Cox regression model of bladder cancer cases diagnosed in Mallorca between 2006 and 2011

	Model 1 Original data set ***n*** = 1619				Model 2 Imputed data set ***n*** = 1840			
	HR	95% CI	***p***	Std. Err.	HR	95% CI	***p***	Std. Err.
Sex	1.04	0.74;1.45	0.810	0.17	1.04	0.74;1.48		
Age	1.07	1.05; 1.07	0.000	0.01	1.05	1.04; 1.07	0.000	0.00
Histology (ref. solid)								
Papillary transitional	0.46	0.31; 0.69	0.004	0.09	0.51	0.35; 0.71	0.000	0.1
Other and unspecified	1.99	1.37; 2.91	0.000	0.38	1.90	1.39; 2.59	0.000	0.3
Stage (ref. stage Ta)								
Stage Tis	3.04	0.95; 9.74	0.060	1.80	3.13	0.94; 10.33	0.030	1.7
Stage I	5.00	2.40; 10.39	0.000	1.86	5.36	2.51; 11.45	0.000	1.9
Stage II	18.94	8.63; 41.55	0.000	7.59	20.95	9.31; 47.11	0.000	7.3
Stage III	37.40	16.34; 85.62	0.000	15.80	37.40	15.85; 88.25	0.000	12.5
Stage IV	84.37	38.64; 184.21	0.000	33.61	72.93	33.21; 160.15	0.000	23.9

Note: *HR* Hazard ratio

## Discussion

Cancer-specific bladder survival at 5 years in Mallorca was 76%, and relative survival was 69%, higher to the unadjusted European average (66.28%) [20], probably because our percentage of non-invasive cancers (Ta and Tis) was high (40.5%).

Survival by stage in bladder cancer varied greatly according to stage, among both non-invasive and invasive cancers. In non-invasive carcinomas, it is probably related with the grade. As far as we know, this is the first study that shows survival by stage in bladder cancer using the UICC 7th edition instead of the simplified classification (localized, regional and distant), which masks important differences in survival under the category of localized. We have observed a different survival between Ta and Tis, as well as a similar survival between Tis and T1. Between stage I and stage II, survival at 5 years halved. Survival for stage IV was very poor, lower than 10%, as found in other studies [7, 8]. The use of multiple imputation for unstaged cases was important in order to not overestimate the survival by stage, as probably happened in other studies [7]. Relative survival was lower than cancer-specific survival, globally and in each stage, as expected according other studies [21, 22].

Apart from stage, age and histology were associated with survival in bladder cancers, but not sex. These cancers are closely related with age. In our study, two of three cases were 65 or more years old, but age was also associated to survival, especially in people older than 74. It is concordant with some studies [4, 7], but not with all of them [8]. Papillary transitional cell carcinoma cases had better survival than solid transitional ones, as expected. The other and unspecified histology category was heterogeneous and showed that the survival of these cases was better than the solid transitional cell cases. All together could add information to the results of a systematic review, which did not find worse prognosis for histological variants [23].

Regarding sex, most studies have found worse survival of bladder cancer in women respect to men, contrary to what happens with other cancers. Differences in stage at the diagnosis, anatomical differences, diagnostic delay, or more accurate diagnosis and treatment in men have been argued to explain such difference in survival [4, 9, 24–26]. Nevertheless, a study has recently observed that women have a less favourable prognosis in bladder cancer only the first 2 years after diagnosis, particularly in a muscle invasive disease [9]. We found worse survival in women in bivariant analysis, but no differences in survival by sex adjusting by age, histology, and stage. Differences in mortality were found after adjusting also by stage, but by simplified classification. So, we add evidence to the no differences of survival by sex in bladder tract cancer.

We opted for cancer-specific survival instead of relative survival, because the Mallorca Cancer Registry has complete access to the cause of death from the Balearic Islands Mortality Registry, and because since 2008, both registries have improved the quality of the data thanks to the access to electronic clinical records from public hospitals and health centres. We are aware that the cancer-specific survival, but also the relative survival, are useful for epidemiologic purposes, but not for the risk communication between clinicians and patients, where the crude mortality, considering competitive risks, is more adequate [27, 28].

Nonetheless, our study is subject to some limitations related to the procedures of the Mallorca Cancer Registry. First, it did not register the grade for non-invasive bladder cancer. Even though there is agreement in that grade is the most important prognostic factor in non invasive bladder cancers [5], there are some discrepancies about which is the optimal classification along with inter observer variability in the pathologist’s grade qualification [29]. In any case, without collecting the grade, the Mallorca Cancer Registry identify part of the high-grade non-invasive bladder cancer, all the solid transitional cases, but we miss the papillary transitional high-grade cases.

Secondly, until 2018, the Mallorca Cancer Registry only registered the first bladder or urinary tract cancer, even if the first was non-invasive and the second was invasive. That means that probably we have missed some multiple (urinary tract and bladder cancers) cases. This has changed and, nowadays, it collects all recurrences.

Finally, Tis may be underreported because some pathologist reports show the diagnosis is transitional papillary carcinoma, but their corresponding complete texts indicate that areas of carcinoma in situ are also viewed. We are aware that, sometimes, we missed this detail.

## Conclusion

Bladder cancer survival vary greatly with stage, among both non-invasive and invasive cases. The percentage of non-invasive cancers is high. Stage is the main factor associated to survival. Age and histology are also associated to survival, but sex has no association.

## Data Availability

All data of the study are available to other authors.

## Abbreviations

DCO: Cases identified only through death certificate
EUROCARE-5: Survival of cancer patients in Europe, 5th edition
HR: Hazar ratio
ICD-O: International Classification of Diseases for Oncology
M: The absence or presence of distant metastasis. It’s a component of TNM
MI: Multiple imputation
MIBC: Muscle-invasive bladder cancer
NMIBC: Non-muscle invasive bladder cancer
N: The absence or presence of regional lymph node metastasis. It’s a component of TNM
N_PT: Pathological or clinical N
REDECAN: Spanish Network of Cancer Registries
T: The extent of the primary tumour
TNM: Classification of malignant tumours. International Union Against Cancer
T_PT: Pathological or clinical T
