# Correction to: Cancer-specific survival by stage of bladder cancer and factors collected by Mallorca Cancer Registry associated to survival

**DOI:** 10.1186/s12885-021-08694-8

**Published:** 2021-08-26

**Authors:** J. Ripoll, M. Ramos, J. Montaño, J. Pons, A. Ameijide, P. Franch

**Affiliations:** 1 Primary Care Research Unit of Mallorca, Balearic Health Service, Palma, Spain; 2 Balearic Islands Health Research Institute (IdISBa), 07120 Palma de Mallorca, Illes Balears Spain; 3 Mallorca Cancer Registry, Balearic Islands Public Health Department, Palma, Spain; 4 University of the Balearic Islands, Palma, Spain; 5 Tarragona Cancer Registry, Cancer Epidemiology and Prevention Service, Sant Joan de Reus University Hospital, IISPV, Reus, Spain

**Correction to: BMC Cancer 21, 676 (2021)**

**https://doi.org/10.1186/s12885-021-08418-y**

Following publication of the original article [1], the authors noticed a missing affiliation for J Ripoll, M Ramos, J Montaño and P Franch. The affiliation is as follows:

- Balearic Islands Health Research Institute (IdISBa), 07120 Palma de Mallorca, Illes Balears, Spain

The affiliations have been correctly published in this correction and the original article [1] has been updated.
